# Preliminary Characterization of a Spray-Dried Hydrocolloid from a High Andean Algae (*Nostoc sphaericum*)

**DOI:** 10.3390/foods11111640

**Published:** 2022-06-01

**Authors:** David Choque-Quispe, Antonieta Mojo-Quisani, Carlos A. Ligarda-Samanez, Miriam Calla-Florez, Betsy S. Ramos-Pacheco, Lourdes Magaly Zamalloa-Puma, Diego E. Peralta-Guevara, Aydeé M. Solano-Reynoso, Yudith Choque-Quispe, Alan Zamalloa-Puma, Ybar G. Palomino-Malpartida, Leidy D. Medina-Quiquin, Aydeé Kari-Ferro

**Affiliations:** 1 Agroindustrial Engineering, Universidad Nacional José María Arguedas, Andahuaylas 03701, Peru; caligarda@unajma.edu.pe (C.A.L.-S.); bsramos@unajma.edu.pe (B.S.R.-P.); 2 Agroindustrial Engineering, Universidad Nacional de San Antonio Abad del Cusco, Cusco 08000, Peru; antonieta.mojo@unsaac.edu.pe (A.M.-Q.); miriam.calla@unsaac.edu.pe (M.C.-F.); lourdes.zamalloa@unsaac.edu.pe (L.M.Z.-P.); 3 Water Analysis and Control Research Laboratory, Universidad Nacional José María Arguedas, Andahuaylas 03701, Peru; deperalta@unajma.edu.pe; 4 Department of Environmental Engineering, Universidad Tecnológica de los Andes, Andahuaylas 03701, Peru; ayma_21@hotmail.com; 5 Department of Environmental Engineering, Universidad Nacional José María Arguedas, Andahuaylas 03701, Peru; ychoque@unajma.edu.pe; 6 Department of Physics, Universidad Nacional de San Antonio Abad del Cusco, Cusco 08000, Peru; alan.zamalloa@unsaac.edu.pe; 7 Department of Chemical Engineering, Universidad Nacional de San Cristóbal de Huamanga, Ayacucho 05000, Peru; ybar.palomino@unsch.edu.pe; 8 Department of Agroindustrial Engineering, Universidad Nacional de San Cristóbal de Huamanga, Ayacucho 05000, Peru; leidy.medina@unsch.edu.pe; 9 Department of Agroecological Engineering and Rural Development, Universidad Nacional Micaela Bastidas, Abancay 03701, Peru; akari@unamba.edu.pe

**Keywords:** spray dried, hydrocolloid, nostoc, stabilizer, zeta potential (ζ)

## Abstract

The search for new natural sources of hydrocolloids with stabilizing, thickening, and good binding capacity, from raw materials that are environmentally friendly and that contribute to the circular economy is a challenge for the food industry. The aim of the study was the preliminary characterization of a spray-dried hydrocolloid from high Andean algae *Nostoc sphaericum*. Four ecotypes of algae from Peruvian high Andean lagoons located above 4000 m were considered. The samples were collected in the period March–April 2021 and were subjected to a spray drying process in an aqueous medium. The characterization showed that the dehydrated nostoc ecotypes presented high protein and carbohydrate content, making it a potential material for direct use as a functional food for humans. The spray-dried product presented good stability for its use as a hydrocolloid, with zeta potential values (ζ), around 30 mV, evidencing the presence of -CO-, -OH, -COO-, and -CH groups, characteristic of polysaccharides, representing 40% of total organic carbon on average, giving it low water activity values and particle size at the nanometric level. Major minerals such as Ca (>277 mg/100 g), Mg (>19.7 mg/100 g), and Fe (>7.7 mg/100 g) were reported. Spray-dried nostoc is a hydrocolloid material with high potential for the food industry, with good nutritional content and techno-functional behavior.

## 1. Introduction

Hydrocolloids are highly hydrophilic substances of high molecular weight, with good water solubility and hydration [1,2], are mainly of biological origins, such as algae, seeds, and tubers, and are made up of polysaccharides including gum arabic, guar gum, carboxymethylcellulose, carrageenan, starch, pectins, and gelatins [3,4], which present different capacity to form gels or act as thickeners in different conditions and at low concentrations, forming colloids with particular structures; due to this property they are widely used in the food industry as additives [5,6].

The current trend in food processing is the use of natural additives as thickeners and stabilizers, with adequate nutritional and techno-functional properties, focusing research on sources of vegetable origin [5,6,7,8,9]; hydrocolloids have recently been extracted from algae, one of them being the Nostocaceae family [3,10,11,12].

In the Andes Mountains, the numerous deposits of lagoons, streams, springs, and in various aquatic environments, it is very common to observe colonies of gelatinous consistency of spherical or lobular shape, bluish-green in color, these are Cyanophyta algae of the *Nostoc sphaericum* type, commonly called murmunta, cushuro, llullucha, crespito, nostoc, yurupa, river grapes, and cochayuyo [13,14,15]. The first botanical report on these algae was in 1892 by Nils Gustaf Lagerheim, who discovered it in Bolivia, Peru, and Ecuador and is used in traditional food in stews and salads or consumed directly [15] due to its high content of fibers, amino acids, proteins, vitamins, and carbohydrates [16,17].

The use of nostoc in obtaining hydrocolloids would reduce the cost of nutritional inputs since these photosynthetic algae do not require complex nutritional needs [10,18,19,20]. Moreover, its production is environmentally friendly and allows the implementation of a circular economy [10].

The use of the hydrocolloid obtained from nostoc in the food industry will be dependent on the physical and chemical properties that it presents; for example, knowing the gelatinization temperature will allow its use in various formulations that are applied in the production of juices, yogurts, and jams, among others [21,22,23,24]; a thermogravimetric analysis would allow knowing the decomposition that it would suffer when used in the baking industry [25,26,27]. Another critical aspect of hydrocolloid additives is their stability, and this is known through knowledge of the zeta potential and the particle size that it presents [28,29].

The extraction methods of hydrocolloids can define the techno-functional, physical, and chemical behavior. Extraction methods are currently being developed to preserve the natural qualities of raw materials [1], avoiding or minimizing the use of secondary substances, as well as other extraction processes; in this sense, the spray-dried method is conservative in terms of initial properties [2,30,31]. This study aimed to preliminarily characterize the spray-dried hydrocolloid of ecotypes of the high Andean algae *Nostoc sphaericum*.

## 2. Materials and Methods

### 2.1. Raw Material

Four nostoc ecotypes (*Nostoc sphaericum*) were collected from high Andean lagoons in the provinces of Cusco and Andahuaylas, Peru, in the period March–April 2021; the description of the lagoons is presented in Table 1.

### 2.2. Determination of Fresh Nostoc Size

Since the nostoc samples are spherical with different sizes, the diameter was measured using a Vernier, and their classification was carried out.

### 2.3. Proximal Analysis of Nostoc Samples

Moisture, protein, ash, fat, soluble fiber, and carbohydrates were determined, following the methodologies proposed by Association of Official Analytical Chemists [32], for nostoc samples dehydrated under vacuum at 10 mbar, 20 °C, for 24 h, in a dryer Binder brand model VD56 (Tuttlingen, Germany). Likewise, the humidity and pH of the fresh nostoc were determined [32].

### 2.4. Nostoc Spray-Dried

The samples were liquefied in a ratio nostoc-distilled water of 1/1, and sieved at 45 microns. The juice obtained was spray-dried in continuous agitation of 100 rpm in a dryer mini spray equipment model B-290, Buchi brand (Flawil, Switzerland). The spray drying process was performed at 100 °C inlet temperature, an air speed of 600 L/s, and a suction speed of 38 m^3^/h.

### 2.5. Total Carbon Determination

To determine the total organic carbon (TOC), 50 mg of nostoc sample dehydrated under vacuum and spray-dried, was placed in ceramic crucibles and covered with fiberglass. It was then taken to the TOC module, Shimadzu brand model TOC–L CSN–SSM 5000A (Kyoto, Japan), with an oxygen flow 150 mL/min; the samples were subjected to 900 °C in the combustion chamber, and the results were reported in triplicate, through the TOC control L V. 1.07 software.

To determine the inorganic carbon (IC), 500 µL of phosphoric acid (1 acid/2 water by volume) was added directly to the sample and placed in the combustion chamber of the apparatus at 200 °C.

### 2.6. Determination of Water Activity (a_w_)

Vacuum dehydrated and spray-dried nostoc samples were taken to a previously calibrated water activity (*a*_w_) determiner, Rotronic brand, model HygroPalm23-AW (Bassersdorf, Switzerland).

### 2.7. Color Determination

The color of fresh, vacuum-dried, and spray-dried nostoc was determined in the CIE L* a* b* space, with criteria: L* luminosity (0 = black and 100 = white), a* and b* chroma (+*a* = red, −*a* = green, +*b* = yellow, and −*b* = blue) [33], the samples were placed on a glass plate and taken to a Konica Minolta colorimeter, model CR-5 (Tokyo, Japan), the readings were performed in reflectance module.

In addition, the color index (IC*) was determined (Equation (1)), which allows color to be expressed in a single numerical datum [34], and is interpreted as follows:

- If IC* −40 to −20, colors range from blue-violet to deep green.
- If IC* −20 to −2, colors range from deep green to yellowish-green.
- If IC* −2 to +2, represents greenish-yellow.
- If IC* +2 to +20, colors range from pale yellow to deep orange.
- If IC* +20 to +40, colors range from deep orange to deep red.

(1) $$IC^{*}=\frac{a^{*}\times 1000}{L^{*}\times b^{*}},$$

### 2.8. IR Analysis of Nostoc

Pressed tablets were prepared with 0.1% of the film in KBr (IR Grade, Darmstadt, Germany). They were brought to the transmission module of the FTIR spectrometer (Fourier-transform infrared spectroscopy), Thermo Fisher (Waltham, MA, USA), Nicolet IS50 model, in a range of 4000 to 400 cm^−1^ with a resolution of 4 cm^−1^.

### 2.9. Thermal Analysis

The thermal stability of the spray-dried nostoc was determined by thermogravimetric analysis (TGA); the samples were loaded in alumina crucibles (Al_2_O_3_) and taken to a TA Instruments brand equipment, model TGA550 (Delaware, CA, USA), with Trios V software, in the range of 20 to 600 °C, with a heating rate of 10 °C/min, and nitrogen supply of 50 mL/min.

### 2.10. Determination of Particle Size and ζ Potential

In total, 4 mg of the sample was dispersed in 5 mL of ultrapure water (neutral pH), stirring at 1000 rpm for 5 min, then ultrasonicated for 10 min at room temperature. An aliquot was taken in a capillary, and the hydrodynamic diameter and size distribution was determined by dynamic light scattering (DLS) using the Nicomp brand equipment, nano ZLS, Z3000 (Massachusetts, MA, USA), with a bandwidth of 33 µsec, and an angle of 90° dispersion; likewise, 2 mL was taken in a polystyrene cell and read in the same equipment, at a laser wavelength of 632.8 nm, a scattering angle of −14.14°, and electric field strength of 5 V/cm, to know the ζ potential.

### 2.11. Mineral Determination

In total, 500 mg of the vacuum-dried and spray-dried nostoc samples were digested at 180 °C for 20 min in an acid medium (12 mL of 65% nitric acid/3 mL of 37.7% hydrochloric acid) gauged with ultrapure water to 50 mL, in a microwave digester, SCP Science brand, MiniWave model (Quebec, QC, Canada). The digested samples were filtered at 0.45 µm and an aliquot was taken for metal quantification in an inductively coupled plasma optical emission spectrometer, Shimadzu brand, model ICP-OES 9820 (Kyoto, Japan). Calibration curves were previously prepared for the metals under study, with a regression coefficient, R^2^ > 0.995. Aliquots were analyzed in axial mode with 10 L/min argon gas flow and 30 s plasma exposure, with a 30 s rinse at 60 rpm between samples.

### 2.12. Statistical Analysis

The results were analyzed through a one-way ANOVA and Tukey’s multiple comparison test at 5% significance.

## 3. Results and Discussion

### 3.1. Fresh Nostoc Size

**Figure 1:**
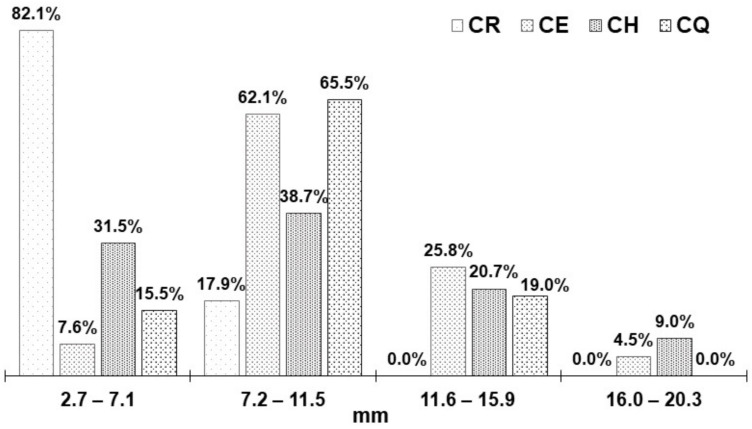
Fresh nostoc diameter.

The fresh nostoc presented variable diameters (*p*-value < 0.05); it was observed that the ecotypes CE, CH, and CQ presented diameters that oscillated between 7.2 to 11.5 mm mostly; 82.1% of the CR ecotype reported lower diameters. In contrast, the CH ecotype reported 9.0% of diameters close to 20.3 mm (Figure 1).

Although the diameter of this type of algae depends on the hydration they receive, this is greater in the rainy season, which frequently occurs in the Peruvian Andes between December and March [8,13,15].

### 3.2. Nostoc Composition

Regarding the pH of fresh nostoc, it was observed that the ecotypes present significant differences (*p*-value < 0.05) (Table 2); CR and CQ show a slight alkaline tendency, while CE and CH are slightly acidic, this pH variation is due to the environment where the algae develop, such as the soil, flora, and fauna of the water body that contains it [3,8,14]. The CE and CH samples were collected from the spring; the others came from lagoons.

Regarding the nutritional composition of dehydrated nostoc (Table 3), it was observed that the majority content is referred to carbohydrates that range between 57.30 to 64.38% (*p*-value < 0.05), mainly composed of polysaccharides (gums and hydrocolloids) [22,35], making these raw materials potential sources for the production of suspension stabilizers [2,4,36,37,38].

Total fat content is reported close to 1.7%, soluble fiber is around 9.0%, while the proteins in the ecotypes are around 24% (*p*-value < 0.05). Algae from lagoon and spring waters are sources of high protein content [8,15,16,17,36,39], in addition to being easily digestible [40], which is why the inhabitants of the high Andean areas of the Andes in South America incorporate it into their diet [15], and in many cases, they substitute sources of protein from animal origin, due to the lack of dairy and meat derivatives.

### 3.3. Total Carbon in Dehydrated and Spray-Dried Nostoc

The structures of proteins, carbohydrates, fats, and fibers are mainly constituted by carbon atoms; the identification of total organic carbon (TOC) would allow evidence of the presence of these constituents in food. Hydrocolloids are made up of carbon chains of polysaccharides with -CO and C-C- groups [11,41,42,43].

**Figure 2:**
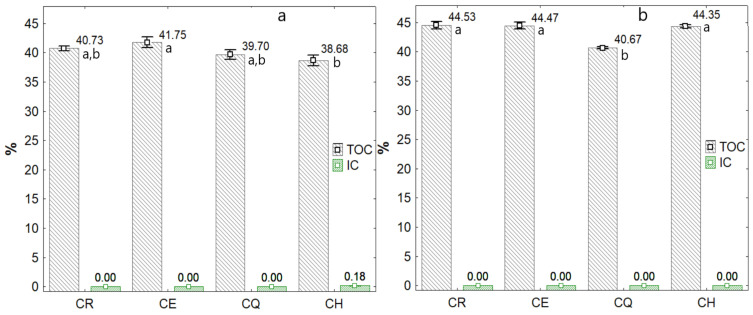
Total carbon in the nostoc, (**a**) dehydrated and (**b**) spray-dried. Different letters indicate significant difference, evaluated with the Tukey test at 5% significance.

The dehydrated nostoc samples reported TOC values of around 40% (Figure 2a), while the spray-dried sample increased to values of 44% (Figure 2b). This increase and net amount in the hydrocolloid should be mainly due to the water-soluble polysaccharides because the spray drying process was carried out from a nostoc-water mixture, although components such as fats, protein, and fibers could be part of the residues, which were retained in the drying chamber of the spray equipment. On the other hand, no significant amounts of inorganic carbon (nondetectable) were observed for dehydrated and spray-dried nostoc, although dehydrated -CH reported 0.18%.

### 3.4. Water Activity

The water activity provides indirect information on the hygroscopic capacity of hydrocolloids due to the active receptor sites of water molecules on their surface. It was observed that the spray-dried nostoc considerably increased a_w_ compared to dehydrated algae for all ecotypes (Table 4); these values suggest a product stable in the environment.

### 3.5. Color

Color is an important sensory attribute in materials, especially if it is used in the food industry [44,45]. It was observed that the luminosity L* or brightness increases significantly in dehydrated and spray-dried nostoc (*p*-value < 0.05) (Table 5), as it happens in vegetables subjected to thermal treatments [46,47], the same way happens for chroma a* with light green tendencies; while b* decreases significantly in the spray-dried hydrocolloid (*p*-value > 0.05, which suggests that it acquires yellow tones [46].

Regarding the color index (CI*), it was observed that the algae in the fresh state showed colors with a tendency to be deep green (Table 5). Values in the range of −20.0 to −2.0 suggest a yellowish-green color [34], as occurs with dehydrated and spray-dried nostoc; this would be mainly due to the conversion of chlorophylls into pheophytins due to the effect of temperature [46,47,48,49].

### 3.6. Infrared Analysis

**Figure 3:**
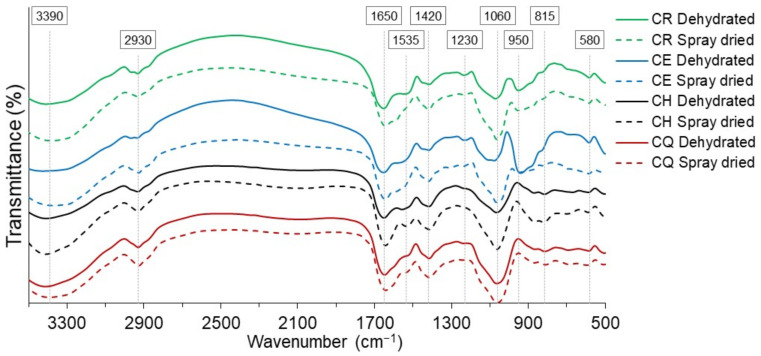
IR spectra for dehydrated and spray-dried nostoc.

The IR spectra of the nostoc samples are shown in Figure 3; an intense peak was observed, around 3390 cm^−1^, indicating the presence of hydroxyl groups, coming mainly from carbohydrates (carboxylate group) and water, with higher intensity for spray-dried samples, and which is related to the activity of the water found. The band at 2900 cm^−1^ is attributed to the stretching vibration of the methyl group of the carbohydrate structure. The peak around 1650 cm^−1^ would correspond to the bending of the adsorbed water, whose intensity increases in the spray-dried materials, which indicates the hydrophilic nature of these samples; likewise, asymmetric stretching vibration of the -COO- corresponding to the carboxylate anions was observed [6].

At 1535 cm^−1^, a slight peak is observed that would correspond to stretching vibration of the -COO- and -NH groups, which is characteristic of amino acids, proteins, and sugars. At 1420 cm^−1^, symmetric stretching of the -COO- corresponding to the carboxylate anion, and methyl groups (-CH_2_) is shown. At 1060 cm^−1^ (Table 6), the presence of functional groups corresponding to polysaccharides and carbohydrates is manifested [11,18,50], with higher intensity for spray-dried samples.

The peaks from 900 to 500 cm^−1^, which are in the “fingerprint” range of the materials, correspond to methylene groups, mainly constituted by the carboxylate anion, and carbohydrates, which are constituents of hydrocolloids. In this sense, a low-intensity spectrum was observed around 580 cm^−1^, corresponding to the asymmetric bending of the O-S-O bond, which corresponds to the absorption due to plant pigments such as chlorophyll a and b [51].

It was clearly observed that the spray-dried nostoc presented higher intensity for the carboxyl, carbonyl, and hydroxyl functional groups, so it would present an anionic character, which is characteristic of hydrocolloids [6,52,53,54,55,56], making it a potential material for use as a food stabilizer [11].

**Table 6:** Fourier transforms infrared (FTIR) spectra for nostoc.

Wavelength (cm^−1^)		Functional Group	Vibration Type	Compound Type	Present in
Range *	In Nostoc				
3700–3100	3390	-NH and -OH	Stretching	Water, secondary amide, and carboxylate	All dehydrated and spray-dried
3000–2800	2930	-CH- aliphatic	Stretching	Methyl groups	All dehydrated and spray-dried
1870–1650	1650	-OH, -COO- asymmetrical, and C=O carbonyl	Bending, stretching	carboxylate anion, ester carbonyl groups	All dehydrated and spray-dried
1600–1500	1535	-COO-, -C(=O)-NH-, C=O, -NH	Stretching	Carboxyl groups, amine	All dehydrated and spray-dried
1500–1300	1420	-CH2, -COO- symmetrical	Stretching	Methyl groups, carboxylate anion	All dehydrated and spray-dried
1300–1200	1230	C-O-C, C-OH, -C-H-	Stretching and bending	Carbohydrates	All dehydrated and spray-dried
1100–920	1060	C-O, C-O-C, C-OH	Stretching	absorption region of polysaccharides	All dehydrated and spray-dried
	950	-OH out of the plane, C-O stretching	bending	Carboxylate anion	CR and CE
	815	-CH- glycosidic	bending	Methylene groups, Glucose	All dehydrated and spray-dried
	580	O=S=O, CHO	bending	Sulfate, Carbohydrates	All dehydrated and spray-dried

* Table is adopted and constructed from references [53,54,55].

### 3.7. Thermal Analysis of Nostoc

**Figure 4:**
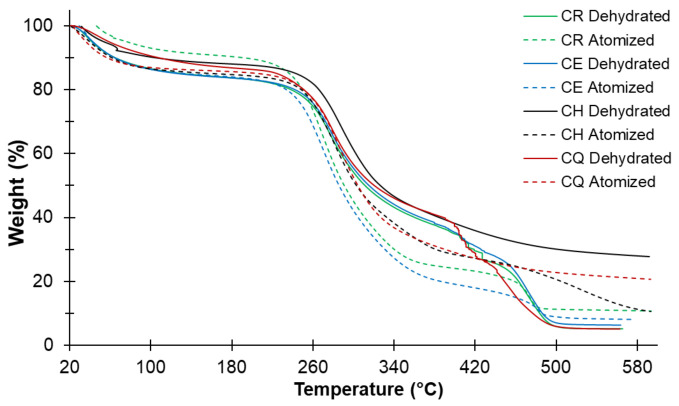
TGA curve for nostoc ecotypes.

The thermal behavior of nostoc, shown in Figure 4, shows three stages of decomposition. The first one is related to the loss of free and bound water [41,57,58], linked to the carbohydrates of the hydrocolloids in the dehydrated and spray-dried nostoc; this stage happens up to around 100 °C, although the spray-dried CE and CH report 85.31 and 78.67 °C, respectively (Table 7). It was observed that the spray-dried CR sample lost less amount of water (7.44%), while the CE, CH, and CQ samples reported water losses of 12.56, 12.19, and 13.02%, respectively. An increase in the thermal stability of spray-dried nostoc was observed for the CR and CQ ecotypes; this would be conditioned to their initial composition (Table 3).

The second stage is related to the decomposition of low molecular weight carbohydrates and peptides due to the depolymerization of their unit molecules; these happen at around 300 °C [57,58,59]. In this same stage, the decomposition of hydrocolloids (series of mono and disaccharide sugars) occurs, due to the breaking of glycosidic bonds, mainly mannose and galactose, of the -C-C, -CO type [41,42], which happens around 200 °C [43,58,60].

It was observed that the spray-dried nostoc samples lose more mass at around 350 °C, being higher for CR and CE (Table 7), which would indicate that these samples have a higher hydrocolloids content due to the fact that they present a prolonged slope in the TGA curve, starting around 200 °C [60] (Figure 4), which confirms the TOC content (Figure 2); this behavior is similar for hydrocolloids such as pectin, xanthan, CMC, and guar gum [4,5,11,58,61,62,63,64].

The third exothermic stage is associated with the decomposition of high molecular weight proteins and polysaccharides (fibers and cellulose), producing CO_2_, NO_2_, and SO_2_, due to the total decomposition of the organic matter until the formation of ashes, polynuclear structures of aromatic carbon, and graphitic [42,57,62,65]. This decomposition stabilizes from approximately 450 °C for the spray-dried samples, reporting mass losses of around 10% for the spray-dried CR, CE, and CQ; for CH, it was 27.06% (Table 7). The dehydrated samples show stability above 500 °C (Figure 4).

### 3.8. Particle Size and ζ Potential

The CQ, CE, and CR ecotypes are classified into three groups, while CH presents two defined groups, according to the NICOMP distribution, observing that the particles with a larger diameter are found in a higher percentage (Table 8). The CR ecotype reported a diameter of 217.3 nm according to the NICOMP distribution and 379.1 ± 254 nm according to the Gaussian distribution.

The fact of presenting more than two peaks would be due to the spray-drying process since the process conditions promote electrostatic and chemical interactions, which generate the formation of larger agglutinated particles; similarly, this behavior would be due to the decomposition of the constituents of the spray-dried nostoc, which when dissolved in water are solubilized [66], being able to find isolated particles of some proteins, fats, and carbohydrates, which present different diameters, similar behavior reported by Cuadros-Moreno et al. [67].

In general, it was observed that spray-dried nostoc is in the nanoparticulate range, with values ranging from 0.9 to 648.7 nm, which would allow better molecular interaction by acting as a hydrocolloid with other substances [2,3,7,26,28,68,69,70,71].

The ζ potential is a parameter that allows measuring the stability of hydrocolloids in solution due to the electrostatic attraction of the constituents and their matrices [72,73]. ζ > 41 mV (absolute value), indicate higher stability in suspension, dispersion, and homogenization; ζ between 21 and 40 mV indicates moderate stability, while ζ < 20 mV would indicate easy agglomeration and sedimentation [29,74,75].

The spray-dried samples showed medium stability at neutral pH (Table 9); this electrostatic attraction is due to the presence of carboxyl, hydroxyl, and carbonyl groups in the spray-dried nostoc, allowing it to hydrate easily and interact with the polar groups of the matrices [76], although the presence of fats and waxy substances could decrease this interaction [28,72,73,77].

### 3.9. Mineral Content in Nostoc

Calcium is the majoritarian mineral, reporting between 186 to 248 mg/100 on a dry basis for the dehydrated nostoc ecotypes (*p*-value < 0.05) (Table 10), and it increases considerably in the spray-dried product.

On the other hand, the mineral content was distributed in the order Mg > Ba > Mg > Na > Fe > Al > K > Zn > Se > Mn, while the other minerals were less than 1 mg/100 g, the same trend was observed in the spray-dried nostoc. The difference in mineral content in the different ecotypes would be due to the composition of the soils surrounding the water sources.

## 4. Conclusions

Dehydrated nostoc ecotypes have high protein and carbohydrate content, which makes them potential for direct use as a functional food. Regarding the spray-dried product, it was observed that it presents good stability for its use as a hydrocolloid in suspensions, with ζ potential values close to 30 mV, due to the presence of -CO, -OH, -COO-, and -CH groups, constituents of polysaccharides, represented around 40% of total organic carbon, giving it low values of water activity, and particle size at the nanometric level. The mineral content showed considerable values of Ca (>277 mg/100 g), Mg (>19.7 mg/100 g), Fe (>7.7 mg/100 g) mainly. Spray-dried nostoc is a hydrocolloid material with high potential for the food industry, as a stabilizer, thickener, and binder, with good nutritional value and techno-functional behavior.

## Figures and Tables

**Table 1:** Nostoc collection coordinates.

Ecotype Code	Source	Community	District	Region	Coordinates		Altitude (m)
					S	W	
CR	Rayanniyoc lagoon	Rayanniyoc	Taray	Cusco	13°28′36″	71°54′23″	3775
CE	Concaja spring	Champimayu	Suycutambo—Espinar	Cusco	15°04′13″	71°44′33″	4851
CQ	Ccopi lagoon	Qquencco	Coya	Cusco	13°22′24″	71°51′06″	4211
CH	Pucccocha spring	Huamanilla	José María Arguedas	Apurímac	13°47′52″	73°17′55″	4251

Where: S, south; W, west; CR, CE, CQ, CH are codes assigned by the authors, for the nostoc ecotypes, of the collection localities.

**Table 2:** pH and humidity of fresh nostoc.

Ecotype					pH			Humidity (%, d.b.)		
	$\bar{x}$	±	SD	*	C.V. (%)	$\bar{x}$	±	SD	*	C.V. (%)
CR	7.57	±	0.12	a	1.59	97.29	±	0.08	a	0.08
CE	6.33	±	0.07	b	1.17	98.23	±	0.05	b	0.06
CQ	7.75	±	0.00	c	0.03	97.94	±	0.06	c	0.06
CH	6.91	±	0.00	d	0.01	98.57	±	0.07	d	0.08

Where $\bar{x}$, arithmetic mean; SD, standard deviation; C.V., coefficient of variability. * Different letters in the column indicate a significant difference, evaluated with the Tukey test at 5% significance.

**Table 3:** Composition of dehydrated nostoc.

Composition (%)			CR					CE					CQ					CH		
	$\bar{x}$	±	SD	*	C.V. (%)	$\bar{x}$	±	SD	*	C.V. (%)	$\bar{x}$	±	SD	*	C.V. (%)	$\bar{x}$	±	s	*	C.V. (%)
Humidity	10.78	±	0.15	a	1.40	9.44	±	0.11	b	1.16	11.30	±	0.11	c	0.97	10.57	±	0.23	a	2.14
Protein	24.70	±	0.05	a	0.19	24.60	±	0.02	a	0.08	17.03	±	0.25	b	1.45	24.01	±	0.12	c	0.50
Fat	1.73	±	0.04	a	2.08	1.79	±	0.02	a	1.12	1.90	±	0.02	b	1.09	1.88	±	0.04	b	1.87
Ash	4.78	±	0.07	a	1.37	5.52	±	0.13	b	2.29	5.16	±	0.11	c	2.16	6.19	±	0.06	d	1.04
Soluble fiber	8.58	±	0.08	a	0.97	8.67	±	0.04	a,b	0.48	9.07	±	0.13	c	1.47	8.84	±	0.11	b,c	1.26
Carbohydrates	57.30	±	0.50	a	0.87	58.52	±	0.10	b	0.16	64.38	±	0.28	c	0.44	57.32	±	0.10	a	0.18

Where $\bar{x}$, arithmetic mean; SD, standard deviation; C.V., coefficient of variability. * Different letters in the row indicate a significant difference, evaluated with the Tukey test at 5% significance.

**Table 4:** Nostoc water activity.

Ecotype		Dehydrated					Spray-Dried			
	$\bar{x}$	±	SD	*	C.V. (%)	$\bar{x}$	±	SD	*	C.V. (%)
CR	0.568	±	0.006	a	1.00	0.628	±	0.004	a	0.55
CE	0.542	±	0.002	b	0.28	0.612	±	0.002	b	0.25
CQ	0.458	±	0.004	c	0.77	0.533	±	0.001	c	0.22
CH	0.437	±	0.004	d	1.00	0.530	±	0.004	c	0.82

Where $\bar{x}$, arithmetic mean; SD, standard deviation; C.V., coefficient of variability. * Different letters in the column indicate a significant difference, evaluated with the Tukey test at 5% significance.

**Table 5:** Nostoc color.

Ecotype		L*				a*				b*				CI*				*	Color	
		$\bar{x}$	±	SD	C.V. (%)	$\bar{x}$	±	SD	C.V. (%)	$\bar{x}$	±	SD	C.V. (%)	$\bar{x}$	±	SD	C.V. (%)		Referential	Fresh Nostoc
CR	Fresh	11.4	±	0.5	4.3	−17.9	±	1.3	7.4	44.9	±	0.9	2.1	−35.0	±	4.9	13.9	<0.05		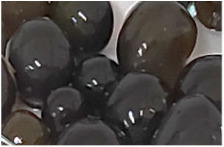
	Dehydrated	40.8	±	2.9	7.1	0.7	±	0.1	10.1	48.2	±	2.4	4.9	0.4	±	0.0	13.5			
	Spray-dried	45.3	±	0.8	1.9	−6.1	±	0.6	9.7	38.3	±	1.9	4.9	−3.5	±	0.4	12.6			
CE	Fresh	17.8	±	0.7	4.1	−20.6	±	2	9.8	52.7	±	0.9	1.7	−22.1	±	1.7	7.5	<0.05		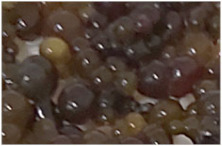
	Dehydrated	24.5	±	0.9	3.5	−9.3	±	0.7	7.3	47.4	±	1.4	3.0	−8.0	±	0.3	3.1			
	Spray-dried	51.5	±	2.7	5.3	−7.5	±	0.7	9.8	36.1	±	2.4	6.5	−4.1	±	0.4	9.1			
CQ	Fresh	32.1	±	1.8	5.7	−9.8	±	0.4	4.4	37.5	±	1.4	3.8	−8.1	±	0.8	9.6	<0.05		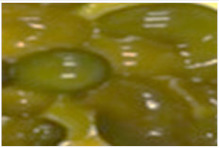
	Dehydrated	43.5	±	3.4	7.9	−4.3	±	0.3	7.0	34.1	±	1.8	5.3	0.3	±	0.0	10.8			
	Spray-dried	53.7	±	0.9	1.6	−7.1	±	0.4	5.4	19.0	±	0.3	1.5	−7.0	±	0.3	4.5			
CH	Fresh	30.1	±	1.4	4.6	−26.1	±	1.1	4.4	36.6	±	2.4	6.5	−23.8	±	2.7	11.4	<0.06		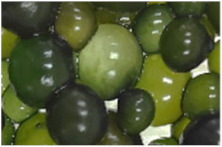
	Dehydrated	35.6	±	2.6	7.2	−21.8	±	2.4	10.9	59.7	±	3.2	5.4	−4.9	±	0.2	4.7			
	Spray-dried	55.3	±	0.4	0.7	−12.1	±	0.4	3.5	32.4	±	0.6	1.9	−6.8	±	0.1	1.5			

Where $\bar{x}$, arithmetic mean; SD, standard deviation; C.V., coefficient of variability. * Evaluated with the Tukey test at 5% significance.

**Table 7:** Lost weight and decomposition temperature.

Ecotype	First Stage		Second Stage		Third Stage		Residue (%)	Max. Weight Loss (%)
	Weight Loss (%)	Temp. (°C)	Weight Loss (%)	Temp. (°C)	Weight Loss (%)	Temp. (°C)		
CR Dehydrated	13.54	101.45	36.53	311.56	39.25	482.09	10.69	89.32
CR spray-dried	7.44	107.96	64.63	349.54	10.50	468.81	17.43	82.57
CE Dehydrated	13.69	101.19	36.51	316.03	39.62	479.30	13.18	86.82
CE spray-dried	12.56	85.31	61.90	347.02	10.93	463.05	14.61	85.39
CH Dehydrated	10.18	108.65	45.39	352.22	14.57	492.14	29.86	70.14
CH spray-dried	12.19	78.67	48.71	318.24	27.06	527.09	12.05	87.95
CQ Dehydrated	13.47	91.63	53.31	370.25	18.31	451.96	14.91	85.09
CQ spray-dried	13.02	98.81	55.25	358.81	10.42	485.27	21.31	78.69

**Table 8:** Particle size (nm) of spray-dried nostoc.

Ecotype	NICOMP Distribution			Gaussian Distribution			
	Peak	Size (nm)	%	$\bar{x}$	±	SD	C.V. (%)
CR	1	11.1	0.9	379.1	±	254.0	67.0
	2	48.8	8.9				
	3	217.3	90.2				
CE	1	16.5	1.3	496.1	±	460.3	92.8
	2	76.7	11				
CH	1	317.2	87.7	454.0	±	269.2	59.3
	2	43.4	4.2				
	3	421.7	95.8				
CQ	1	22.1	1.3	413.8	±	257.4	62.2
	2	121.6	15.1				
	3	648.7	83.6				

Where $\bar{x}$, arithmetic mean; SD, standard deviation, C.V., coefficient of variability.

**Table 9:** ζ potential of spray-dried nostoc.

Ecotype	ζ Potential (mV)	Viscosity Solution (cP)	Sample Temperature (°C)	pH Solution
CR	−30.88	0.933	23	Neutral
CE	−27.17	0.933	23	Neutral
CH	−27.14	0.933	23	Neutral
CQ	−28.96	0.933	23	Neutral

**Table 10:** Metals in dehydrated and spray-dried nostoc.

**Metal**	**Wavelength (nm)**	CR				CE				CH				CQ			
		Dehydrated															
		$\bar{x}$	**±**	**SD**	*	$\bar{x}$	±	SD	*	$\bar{x}$	±	SD	*	$\bar{x}$	±	SD	*
Al	394.403	8.340	±	0.010	a	5.163	±	0.006	b	10.240	±	0.010	c	15.730	±	0.066	d
As	189.042	1.930	±	0.022	a	2.377	±	0.024	b	4.414	±	0.072	c	3.793	±	0.026	d
Ba	389.178	19.435	±	0.233	a	13.235	±	0.059	b	4.492	±	0.023	c	65.107	±	0.221	d
Be	313.042	0.001	±	0.000	a	0.001	±	0.000	b	0.001	±	0.000	c	0.001	±	0.000	d
Ca	317.933	186.559	±	1.733	a	248.226	±	0.577	b	221.790	±	0.289	c	212.956	±	0.362	d
Cd	214.438	0.140	±	0.003	a	0.170	±	0.001	b	0.320	±	0.001	c	0.299	±	0.001	d
Cr	267.716	0.086	±	0.001	a	0.078	±	0.001	b	0.149	±	0.001	c	0.221	±	0.003	d
Cu	324.754	0.055	±	0.001	c	0.053	±	0.001	c	0.184	±	0.001	a	0.170	±	0.005	b
Fe	239.562	8.841	±	0.047	a	2.394	±	0.006	b	8.354	±	0.060	c	14.495	±	0.066	d
K	766.490	4.420	±	0.061	a	18.447	±	0.100	b	60.847	±	0.095	c	34.463	±	0.015	d
Mg	285.213	25.996	±	0.289	a	30.430	±	0.153	b	48.996	±	0.058	c	32.457	±	0.001	d
Mn	257.610	1.040	±	0.006	a	1.103	±	0.001	b	1.723	±	0.010	c	3.770	±	0.011	d
Na	588.995	14.247	±	0.176	a	6.213	±	0.039	b	13.380	±	0.055	c	16.650	±	0.004	d
Ni	341.476	0.017	±	0.003	c	0.010	±	0.000	c	0.543	±	0.006	a	0.367	±	0.032	b
Pb	220.353	0.507	±	0.015	a	0.577	±	0.006	b	1.183	±	0.006	c	1.083	±	0.038	d
Se	203.985	1.723	±	0.050	a	2.053	±	0.032	b	4.137	±	0.025	c	3.647	±	0.055	d
V	311.071	0.027	±	0.001	b	0.022	±	0.001	c	0.087	±	0.002	a	0.083	±	0.002	a
Zn	213.856	0.197	±	0.002	a	0.362	±	0.002	b	0.268	±	0.002	c	0.301	±	0.002	d
		Spray-dried															
Al	394.403	9.610	±	0.000	a	6.027	±	0.006	b	6.420	±	0.020	c	11.677	±	0.058	d
As	189.042	2.732	±	0.018	a	2.597	±	0.036	b	2.532	±	0.050	b,c	2.452	±	0.038	c
Ba	389.178	34.790	±	0.057	a	20.528	±	0.060	b	2.307	±	0.006	c	49.723	±	0.345	d
Be	313.042	0.001	±	0.000	a	0.001	±	0.000	b	0.002	±	0.000	c	0.001	±	0.000	d
Ca	317.933	283.248	±	0.578	a	277.241	±	0.576	b	306.582	±	1.000	c	271.915	±	2.310	d
Cd	214.438	0.216	±	0.002	a	0.199	±	0.002	b	0.181	±	0.001	c	0.190	±	0.002	d
Cr	267.716	0.139	±	0.001	a	0.077	±	0.001	b	2.436	±	0.010	c	0.104	±	0.001	d
Cu	324.754	0.122	±	0.002	b	0.087	±	0.001	c	0.141	±	0.001	a	0.122	±	0.001	b
Fe	239.562	12.555	±	0.058	a	2.754	±	0.011	b	20.621	±	0.100	c	8.755	±	0.035	d
K	766.490	8.423	±	0.055	c	26.530	±	0.154	b	27.890	±	0.252	a	26.523	±	0.251	b
Mg	285.213	43.240	±	0.116	a	38.173	±	0.100	b	23.407	±	0.152	c	19.774	±	0.201	d
Mn	257.610	1.361	±	0.001	a	1.160	±	0.000	b	1.474	±	0.006	c	1.917	±	0.011	d
Na	588.995	28.430	±	0.102	a	8.863	±	0.039	b	6.130	±	0.064	c	10.796	±	0.122	d
Ni	341.476	0.280	±	0.000	a	0.093	±	0.006	b	2.183	±	0.015	c	0.257	±	0.006	d
Pb	220.353	0.679	±	0.011	b	0.701	±	0.010	b	0.609	±	0.008	c	0.725	±	0.003	a
Se	203.985	2.650	±	0.036	b	2.347	±	0.042	c	2.860	±	0.040	a	2.277	±	0.029	c
V	311.071	0.050	±	0.001	a	0.032	±	0.001	b	0.022	±	0.001	c	0.045	±	0.001	d
Zn	213.856	0.763	±	0.002	a	0.176	±	0.001	b	0.289	±	0.002	c	0.345	±	0.002	d

Where $\bar{x}$, arithmetic mean; SD, standard deviation; C.V., coefficient of variability. * Different letters in the row indicate a significant difference, evaluated with the Tukey test at 5% significance.

## Data Availability

The data presented in this study are available in this same article.
